# Cytokine Activation Reveals Tissue-Imprinted Gene Profiles of Mesenchymal Stromal Cells

**DOI:** 10.3389/fimmu.2022.917790

**Published:** 2022-07-18

**Authors:** Danielle M. Wiese, Catherine A. Wood, Barry N. Ford, Lorena R. Braid

**Affiliations:** ^1^ Aurora BioSolutions Inc., Medicine Hat, AB, Canada; ^2^ Defence Research and Development Canada Suffield Research Centre, Casualty Management Section, Medicine Hat, AB, Canada; ^3^ Simon Fraser University, Department of Molecular Biology and Biochemistry, Burnaby, BC, Canada

**Keywords:** mesenchymal stromal cells (MSC), TNF-α, IL1-β, IFN-γ, licensing, activation, transcriptome, gene profiles

## Abstract

Development of standardized metrics to support manufacturing and regulatory approval of mesenchymal stromal cell (MSC) products is confounded by heterogeneity of MSC populations. Many reports describe fundamental differences between MSCs from various tissues and compare unstimulated and activated counterparts. However, molecular information comparing biological profiles of activated MSCs across different origins and donors is limited. To better understand common and source-specific mechanisms of action, we compared the responses of 3 donor populations each of human umbilical cord (UC) and bone marrow (BM) MSCs to TNF-α, IL-1β or IFN-γ. Transcriptome profiles were analysed by microarray and select secretome profiles were assessed by multiplex immunoassay. Unstimulated (resting) UC and BM-MSCs differentially expressed (DE) 174 genes. Signatures of TNF-α-stimulated BM and UC-MSCs included 45 and 14 new DE genes, respectively, while all but 7 of the initial 174 DE genes were expressed at comparable levels after licensing. After IL-1β activation, only 5 of the 174 DE genes remained significantly different, while 6 new DE genes were identified. IFN-γ elicited a robust transcriptome response from both cell types, yet nearly all differences (171/174) between resting populations were attenuated. Nine DE genes predominantly corresponding to immunogenic cell surface proteins emerged as a BM-MSC signature of IFN-γ activation. Changes in protein synthesis of select analytes correlated modestly with transcript levels. The dynamic responses of licensed MSCs documented herein, which attenuated heterogeneity between unstimulated populations, provide new insight into common and source-imprinted responses to cytokine activation and can inform strategic development of meaningful, standardized assays.

## Introduction

Mesenchymal stromal cells (MSCs) are potential therapeutics for numerous clinical indications, particularly those that exploit the immunomodulatory properties of the cells. Recent clinical advances in MSC-based therapeutics have revealed a need for standardized tests that correlate predictive markers of immunomodulation, regeneration and homing properties with therapeutic efficacy (1–5). Robust and validated potency assays are required by regulatory authorities to fulfill performance criteria for advanced clinical trials and eventual product approval (1, 2, 6). Comparison of stimulus-provoked biomarkers from cytokine-activated MSCs versus unstimulated MSC controls has been proposed as predictive metrics of therapeutic potential (7, 8). Measured biomarkers, or mechanism-of-action surrogates, could include cell surface markers, secreted proteins and expressed transcripts.

MSCs are progenitor cells that reside in the stroma of most tissues and organs. Although MSCs derived from various tissues share core properties and were originally believed to be comparable, source-specific distinctions between MSC populations are increasingly reported. Tissue and donor origin have been linked to unique transcriptome (9–13), secretome (14, 15) and surfaceome (15–18) profiles, as well as variable therapeutic potential (12, 19, 20). Cell isolation and cultivation protocols (reviewed in (4, 5, 21, 22)), including media formulation, can also influence these properties (23, 24). This heterogeneity complicates development of standardized potency assays.

Many studies have explored the biological responses of culture-adapted MSCs to specific inflammatory stimuli, both as a predictor of their response after administration for inflammatory diseases and conditions, and to pre-activate or prime the cells to enhance their therapeutic potential (12, 17, 25–29). There are substantial data describing differences between culture adapted MSCs from different sources, and studies comparing unstimulated and activated counterparts. However, molecular data describing the biological profiles and properties of activated MSCs derived from different origins and donors are limited. Comparative studies addressing this topic have focused on relatively few, select immune-modulatory transcripts, cell surface markers, secreted factors or functions (16, 30–32).

Here we undertook a transcriptome profiling approach to compare the responses of umbilical cord-derived (UC) and bone marrow-derived (BM) MSCs to 3 common inflammatory mediators that activate MSCs via independent signalling pathways. Three representative donor populations, balanced for sex and *in vitro* age, from each MSC source were included to generate a heterogeneous data set. MSC polarization as a result of cytokine activation was assessed at the level of gene expression by microarray, and by secreted output of a panel of cytokines and growth factors. We postulated that licensing of cultured MSCs by pro-inflammatory signals might drive MSCs with underlying differences towards a synchronized phenotype or amplify their differences to produce more distinct cell populations. Understanding these dynamics will enable rational development of robust, standardized MSC potency assays employing activation and functional polarization strategies.

## Materials and Methods

### Cell Source

High quality MSC populations were obtained from commercial sources and qualified by tri-lineage differentiation and cell surface marker profiles as previously described (33) in accordance with internationally accepted minimal criteria (34). UC-MSCs (35) cryopreserved at mean population doubling level (mPDL) 10, were provided by Tissue Regeneration Therapeutics (TRT), Inc. (Toronto, Canada). UC-MSCs are isolated from the perivascular Wharton’s jelly (36) of healthy term (>37 weeks) umbilical cords delivered by C-section, and cultivated in Mesenchymal Stem Cell Growth Media – Chemically Defined™ (MSCGM-CD™; Lonza, Walkersville, MD). BM-MSCs from donors 18-30 years of age, cryopreserved at mPDL 9, were obtained from RoosterBio Inc. (Frederick, MD).

### Cell Culture

For cell expansion, MSCs were seeded at a density of 1333 cells/cm^2^ in RoosterNourish™-MSC-XF (RoosterBio Inc.). Cells were incubated at 37° C, 5% CO_2_ with media changes every 3-4 days and passaged once the monolayer reached 70-80% confluence. Culture media was aspirated and cells washed with DPBS^-/-^ (ThermoFisher Scientific, Waltham, MA), then enzymatically detached using TrypLE™ Select (ThermoFisher Scientific, Waltham, MA). Once cells were dissociated, an equal volume of media was added to the suspension and cells were counted with a Millipore Scepter cell counter using 60 µm probes (MilliporeSigma, Billerica, MA). Cells were centrifuged at 149 x *g* for 5 minutes, and the cell pellet re-suspended in fresh media and seeded in new culture vessels at a density of 1333 cells/cm^2^.

### Cytokine Stimulation

Three donor populations of UC-MSCs and BM-MSCs (2 male and 1 female each) were tested independently, in 3 replicate experiments. MSCs at passage 4 (mPDL 14.6-16.5) were seeded in 25 cm^2^ flasks at a density of 13 333 cells/cm^2^ in 3ml RoosterNourish™-MSC-XF and allowed to adhere overnight. After 18 hours, nearly all cells had adhered and exhibited a normal fibroblastic morphology, generating a monolayer with ~70% confluence. Purified recombinant human cytokines (PeproTech, Inc., Rocky Hill, NJ) were diluted in protein-free (PF) RoosterBasal™-PF media (RoosterBio Inc.) at the following concentrations: TNF-α (50 ng/ml), IL-1β (80 pg/ml) and IFN-γ (50 ng/ml). Complete media was removed from the monolayers and replaced with 2 ml of cytokine solution. Unstimulated cultures received 2 ml of RoosterBasal™-PF without cytokine supplements. To quantify media background, 2 ml of unsupplemented or cytokine-supplemented PF media was added to 25 cm^2^ flasks. All flasks were incubated at 37°C, 5% CO_2_ for 24 hours.

After 24 hours, conditioned media (CM) was collected and stored in small aliquots at -80°C until analysis. To collect mRNA, cells were enzymatically detached from the culture vessel and pelleted by centrifugation as described above, then re-suspended in 0.5 ml of RNAprotect^®^ Cell Reagent (Qiagen, Germantown, MD) for short-term storage at -80°C.

### RNA Extraction

Cell pellets from technical replicates (n = 3 for each donor population) were combined to produce a single RNA sample. RNA was extracted using Qiagen RNeasy^®^ Plus Mini Kits (Qiagen) according to manufacturer’s recommendations. 20 µl of 2 M β-mercaptoethanol was added per ml of kit buffer (RLT buffer) and 600 µl RLT buffer was used for each sample. RNA concentration and purity were assessed in 96 well plates using a BioTek Synergy HT microplate reader (BioTek Instruments Inc., Winooski, VT) and Gen5 2.05 software with path length correction.

### Microarray Processing

RNA expression was analyzed using GeneChip™ U133A 2.0 arrays (Affymetrix, ThermoFisher Scientific) as previously described [28]. Briefly, RNA was amplified and labeled using GeneChip™ 3’ IVT Plus kits (Affymetrix, ThermoFisher Scientific), and hybridized to arrays for 16 hours at 45°C, 60 RPM in a GeneChip™ Hybridization Oven 640 (Affymetrix, ThermoFisher Scientific). Chips were scanned using a GeneChip™ 3000 7G Scanner (Affymetrix, ThermoFisher Scientific) and the resulting images reviewed for quality in GeneChip™ Control Console Viewer.

### Gene Expression Data Analyses

24 microarray .CEL files were analyzed using Bioconductor (37) packages in R version 3.4.2 (38). Pre-processing (GCRMA background adjustment, normalization, log_2_ expression matrix output) was performed using gcrma (39) and Affymetrix U133A 2.0 probe affinity data. Data quality was interrogated using simpleaffy (39–41) following GeneChip™ guidelines (42). To minimize batch influences on data collected, MSC type and cell donor sex were equally distributed (with the exception of 1/24) amongst two culture and stimulation periods, and three RNA extraction and microarray processing batches. Nevertheless, any batch effects were explored by evaluating all BatchQC (43) metrics with and without ComBat (44) adjustment, and subsequently the data was left unadjusted. Using genefilter (45), pre-processed probe sets were filtered to only include those that were expressed greater than 2-fold above background signal on at least 3 arrays; 3 is the smallest number of arrays assigned to a condition (cytokine stimulation within a MSC type). Sixty-two percent of probe sets were included for differential expression analysis.

Limma (46) was used for linear modeling of comparisons and differential expression assessment. Comparisons of resting MSC types, resting versus activated UC-MSCs and BM-MSCs separately, and activated MSC types utilized TREAT (47) to simultaneously select significantly differentially expressed (DE) probe sets having both a Benjamini-Hochberg (48) false discovery rate (FDR)-adjusted p-value (p) of <0.05 and a >2-fold change. Annotation information was accessed through AnnotationDbi (49, 50) and heat maps were created using ComplexHeatmap (51).

Functional analysis was carried out using DAVID (52, 53) by querying DE gene lists’ Entrez identifiers for enriched (FDR 5%) Gene Ontology (GO) Biological Process terms (54, 55) and Kyoto Encyclopedia of Genes and Genomes (KEGG) pathways (56, 57). All non-specific probe sets were addressed by eliminating included Entrez identifiers of read-through transcripts, microRNAs and additional members of the same gene family. The data have been deposited in NCBI’s Gene Expression Omnibus (GEO) database (58, 59), available through accession number GSE129165.

### Reverse Transcription-Quantitative Polymerase Chain Reaction and Analysis

RT-qPCR was performed in triplicate wells using Quantinova SYBR green kits (Qiagen) read by CFX Connect Real-Time System (Bio-Rad, Hercules, CA). Sequences for *ACTB*, *CXCL12*, *GAPDH*, *HLA-DRA*, *IDO1*, *PTGS2, TNFAIP6* and *YWHAZ* primers (MilliporeSigma) are listed in **Supplementary Figure 1A**. Non-detects, due to failure to amplify by cycle 40, had C_q_ values imputed using R packages HTqPCR (60) and nondetects (61); valid undetected values (imputed value <0 across replicates) were subsequently substituted with C_q (max)_+1 (62). *ACTB*, *GAPDH* and *YWHAZ* were used as reference genes for normalization due to greatest stability amongst RefFinder (63) ranking of Reference Genes H96 PrimerPCR Pathway Plate (Bio-Rad) results (data not shown). Relative expression was calculated using 2^Cq(reference gene mean)-Cq(target)^ (64).

### Multiplex Immunoassay

A subset of inflammatory mediators was quantified in CM samples using commercially available multiplex immunoassays. Test samples were thawed at 4°C and used within 24 hours; any remaining sample was discarded. Each of three 25 cm^2^ flask replicate samples per MSC donor were analyzed by Bio-Plex Pro™ (Bio-Rad) Human Cytokine Panel Group I 27-plex and Human TGF-β 3-plex kits, run undiluted and at a final dilution of 1:5 with 0.625% BSA respectively. The observed concentration of samples was exported from Bio-Plex Manager™ 6.1 software and normalized to concentration (pg/ml) per one million cells at CM collection.

### Multiplex Immunoassay Statistical Analysis

The mean analyte concentrations for each MSC donor were used to compare unstimulated MSC types, resting versus licensed UC-MSCs and BM-MSCs separately, and licensed MSC types. Statistical significance was determined by multiple t-tests since the range of analyte concentrations across the assay covered several orders of magnitude. An FDR-adjusted p<0.05 was deemed statistically significant. All analysis was performed using GraphPad Prism version 7 (GraphPad Software, La Jolla, CA).

## Results

### Unstimulated UC and BM-MSCs Differentially Express Immune- and Inflammation-Related Genes

**Figure 1:**
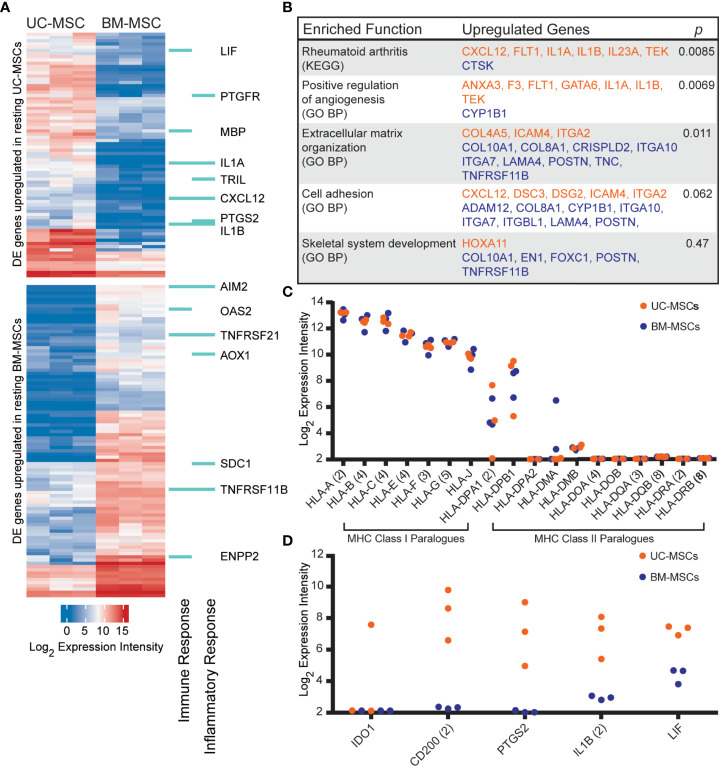
The transcriptomes of unstimulated UC and BM-MSCs exhibit significant differences. **(A)** Seventy-six genes are preferentially expressed by UC-MSCs, while 98 genes are enriched in BM-MSCs. A subset are components of immune and inflammatory responses, as specified by GO. **(B)** Few DE genes exhibit functional co-registration. Genes DE by UC (orange) and BM (purple) MSCs contribute to few enriched (p<0.05) functional annotations. **(C)** UC and BM-MSCs similarly express MHC-I paralogues and lack most MHC-II paralogues. **(D)** Key immunomodulatory genes are more highly expressed in resting UC-MSCs. BM, bone marrow; DE, differentially expressed; GO BP, Gene Ontology Biological Process annotation; KEGG, Kyoto Encyclopedia of Genes and Genomes pathway annotation; MSC, mesenchymal stromal cell; UC, umbilical cord.

The transcriptome profiles of unstimulated UC and BM-MSCs were first established. Of 14,500 assayed genes, 174 exhibited significant expression differences between MSC types. UC-MSCs solely expressed 30 genes, and significantly higher levels of 46 genes compared to BM-MSCs (**Figure 1A**; **Table S1**); few of these differentially expressed (DE) genes could be connected by function or pathway. Several genes contribute to enrichment of the rheumatoid arthritis KEGG pathway and positive regulation of angiogenesis GO function (**Figure 1B**). BM-MSCs uniquely expressed 46 genes, and 52 genes at significantly higher levels than UC-MSCs (**Figure 1A**; **Table S1**). Subsets of these DE genes are linked to extracellular matrix organization, cell adhesion and skeletal system development GO functions (**Figure 1B**). Other DE genes between UC and BM-MSCs included various collagens, phospholipase, integrin subunit, sialyltransferase enzyme and solute carrier family member transcripts (**Table S1**).

DE genes linked to GO immune and/or inflammatory responses were comparably enriched in UC-MSCs (8 genes) and BM-MSCs (7 genes) (**Figure 1A**). Expression of other important immune-related genes was also interrogated (**Figures 1C, D**). Both MSC types expressed major histocompatibility complex (MHC) class I chain paralogues at moderate to high levels and did not express MHC class II paralogues except for *HLA-DPA1* and *HLA-DPB1* (**Figure 1C**). *HLA-DMA* was detected in 2 BM-MSC populations but was only substantial in 1. (**Figure 1C**). The metabolic immunomodulatory gene *IDO1* was detected in only 1 unstimulated UC-MSC population (**Figure 1D**). *CD200*, a disputed surrogate marker for immunosuppression (2, 16), and *PTGS2* (COX-2), which preferentially produces the T-cell inhibitor PGE2 in MSCs (17, 65), were significantly expressed by UC-MSCs but not detected in BM-MSCs (**Figure 1D**). *IL1B* and *LIF*, purportedly linked to the superior immunomodulatory performance of native UC-MSCs compared to BM-MSCs (66), were expressed significantly more by UC-MSCs (**Figures 1A, D**; **Table S1**).

Next, MSCs were strategically activated with inflammatory mediators to assess the continuity of their gene expression and secreted responses to controlled stimulation. TNF-α is a multi-functional inflammatory mediator implicated in numerous signalling and functional pathways. IL-1β is a potent low-abundance cytokine with activity more refined to acute injury or infection, while IFN-γ stimulates the innate and adaptive immune responses to pathogens. MSCs were dosed with a physiologically relevant amount of cytokine, estimated from circulating or local levels reported in clinical samples (67, 68).

### TNF-α Evokes Source-Specific Gene Profiles Related to Inflammation and Immunity

**Figure 2:**
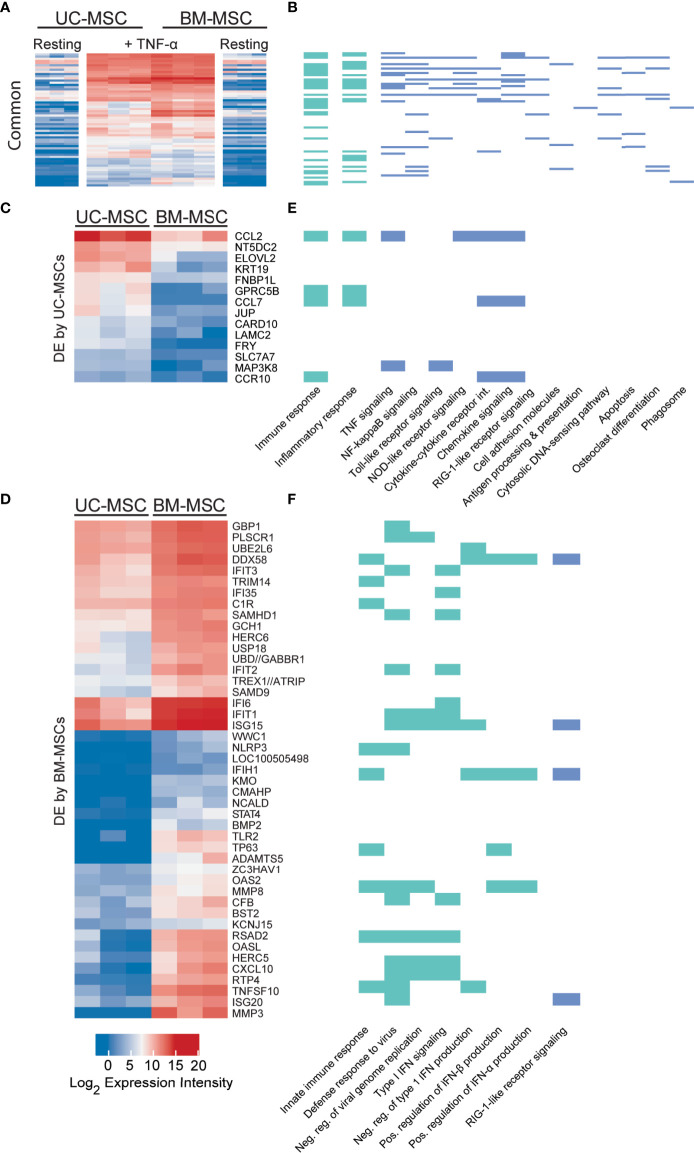
TNF-α resolves all but 7 differentially expressed genes in unstimulated MSCs and evokes source-specific activation signatures. **(A)** Sixty-one genes are activated to comparable levels after licensing of UC and BM-MSCs by TNF-α. **(B)** Most of these genes are linked to enriched GO Biological Process terms are indicated in (green), and enriched KEGG pathways (blue (p<0.05) related to immunity and inflammation. **(C)** Thirteen genes are upregulated in TNF-α licensed UC-MSCs statistically higher than activated BM-MSCs. **(D)** Forty-five genes are statistically upregulated in DE genes TNF-α licensed BM-MSCs. **(E)** UC-MSCs preferentially express genes linked to immunity, inflammation and cytokine and chemokine signaling, while **(F)** BM-MSCs increase expression of genes linked to innate immune function and Type I IFN signalling. BM, bone marrow; DE, differentially expressed; MSC, mesenchymal stromal cell; UC, umbilical cord.

Sixty-one genes were upregulated to comparable levels between TNF-α activated UC- and BM-MSCs (**Figure 2A**; **Table S2**), most of which were linked to GO immune and inflammatory processes (**Figure 2B**). Only 7 of the 174 genes that were DE between unstimulated MSC types remained significantly DE between TNF-α activated MSC types (**Table 1**). However, TNF-α evoked fifty-nine newly significantly DE genes between TNF-α licensed UC and BM MSCs (**Table 1**; **Figures 2C, D**). Fourteen genes preferentially expressed by TNF-α polarized UC-MSCs are primarily involved in inflammation or immunity (*CCL2, CCL7*, *CCR10*, *GPRC5B*, *MAP3K8*; **Figures 2C, E**), or structure or adhesion (*FNBP1L*, *FRY*, *JUP*, *KRT19*, *LAMC2*). Forty-five of 59 DE genes more abundant in BM-MSCs are linked to the following enriched GO functions: innate immunity, viral defense, including negative regulation of viral genome replication and type I interferon (IFN) signalling, with negative regulation of type I IFN production and positive regulation of IFN-α and IFN-β production (**Figures 2D, F**). Notably, none of the 14 genes DE by UC-MSCs are involved in these functions (**Figure 2E**). Taken together, the differences between resting UC and BM-MSCs are attenuated by TNF-α stimulation, accompanied by the introduction of new source-specific transcriptome signatures (**Table 1**), most notably the preferential activation of Type I IFN signalling in BM-MSCs.

**Table 1:** Statistically significant differences between UC-MSCs and BM-MSCs after cytokine activation.

Different:	In resting and licensed cells (unchanged by activation)	After licensing only (changes induced by activation)
**+ TNF-α**		
↑ in UC-MSCs	*MBP*, *ST6GALNAC5*, *TEK*	*CARD10, CCL2, CCL7, CCR10, ELOVL2, FNBP1L, FRY, GPRC5B, JUP, KRT19, LAMC2, MAP3K8, NT5DC2, SLC7A7*
	
↑ in BM-MSCs	*CPM*, *IFI44L*, *KRTAP1-1*, *MMP13*	*ADAMTS5, BMP2, BST2, C1R, CFB, CMAHP, CXCL10, DDX58, GBP1, GCH1, HERC5, HERC6, IFI35, IFI6, IFIH1, IFIT1, IFIT2, IFIT3, ISG15, ISG20, KCNJ15, KMO, LOC100505498, MMP3, MMP8, NCALD, NLRP3, OAS2, OASL, PLSCR1, RSAD2, RTP4, SAMD9, SAMHD1, STAT4, TLR2, TNFSF10, TP63, TREX1//ATRIP, TRIM14, UBD//GABBR1, UBE2L6, USP18, WWC1, ZC3HAV1*
	IL-10, IL-12, IL-13, VEGF	IL-7, RANTES
**+ IL-1β**		
↑ in UC-MSCs	*IL1A*, *PTGS2*	*GPRC5B*, *MAP3K8*
	
↑ in BM-MSCs	*B3GALT2*, *COL10A1*, *KRTAP1-1*	*BST2*, *MMP3*, *STAT4*, *WISP1*
	IL-10, IL-12, IL-13, VEGF	IL-7, IP-10
**+ IFN-γ**		
↑ in UC-MSCs	*CCNA1*, *CXCL12*	*RIMS2*
	
↑ in BM-MSCs	*BAALC*	*ACKR4*, *CMAHP*, *HLA-DQA1//HLA-DQA2*, *HLA-DQB1*, *HLA-DRA*, *KCTD14*, *OASL*, *TLR2*
	IL-13	

↑, DE by specified MSC type. UC, umbilical cord-derived MSCs. BM, bone marrow-derived MSCs. //, non-specific probe set. Italicized font denotes a transcript; regular font denotes a soluble protein.

### IL-1β Drives UC and BM-MSCs Towards A Similar Transcriptome Profile With Few Signature Differences

**Figure 3:**
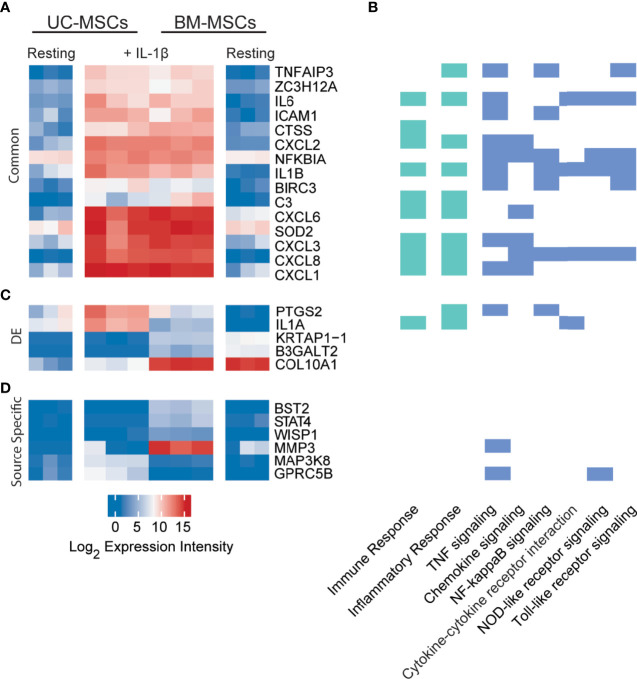
Expression profiles of IL-1β licensed UC and BM-MSCs include 15 common genes and 6 source-imprinted genes. **(A)** Fifteen genes are significantly upregulated to comparable levels in UC and BM-MSCs activated by IL-1β. **(B)** Genes responsive to IL-1β are involved in many immune and inflammatory processes, as determined by GO (green; see complete enriched list in **Table S4**), as well as 6 enriched KEGG pathways (blue) (p<0.05). **(C)** Only 5 of the 74 genes DE between unstimulated UC and BM-MSCs are still expressed at significantly levels after dosing with IL-1β. **(D)** Four genes are specifically upregulated in IL-1β licensed BM-MSCs, while 2 genes are specifically upregulated in IL-1β activated UC-MSCs. BM, bone marrow; DE, differentially expressed; IL, interleukin; MSC, mesenchymal stromal cell; UC, umbilical cord.

Only 33 genes were significantly responsive to IL-1β priming, 15 of which increased in both MSC types to comparable expression intensities (**Figure 3A** and **Table S3**). These genes have defined roles in immune and inflammation responses. KEGG pathway analysis of all responsive genes revealed enrichment for TNF signaling (12 genes), chemokine signaling (8 genes), NF-κB signaling (8 genes), cytokine-cytokine receptor interaction (5 genes), NOD-like receptor signaling (5 genes) and Toll-like receptor signaling (6 genes) (**Figure 3B**). Top enriched GO functions included inflammatory response (13 genes), immune response (12 genes) and chemotaxis (6 genes) (**Figure 3B** and **Table S4**).

Interestingly, only 5 of the 174 DE genes (*KRTAP1-1, B3GALT2, COL10A1*, *PTGS2* and *IL1A*) remained significantly different between IL-1β licensed UC and BM-MSCs (**Table 1** and **Figure 3C**), although the fold-difference was reduced. Six new DE genes emerged following IL-1β dosing (**Table 1** and **Figure 3D**). *BST2, STAT4, WISP1, MMP3, MAPK3K8* and *GPRC5B* are either not expressed or expressed at similarly modest intensities in unstimulated MSCs. After IL-1β exposure, these genes were upregulated in only 1 or the other MSC type, resulting in unique signatures between MSC types (**Figure 3D**). The signature of IL-1β polarized UC-MSCs includes *MAP3K8* and *GPRC5B*, while polarized BM-MSCs uniquely express *BST2, STAT4, WISP1* and *MMP3* (**Table 1**).

### Source-Specific Transcriptome Profiles of IFN-γ Licensed UC and BM-MSCs Are Linked to Immune Processes

**Figure 4:**
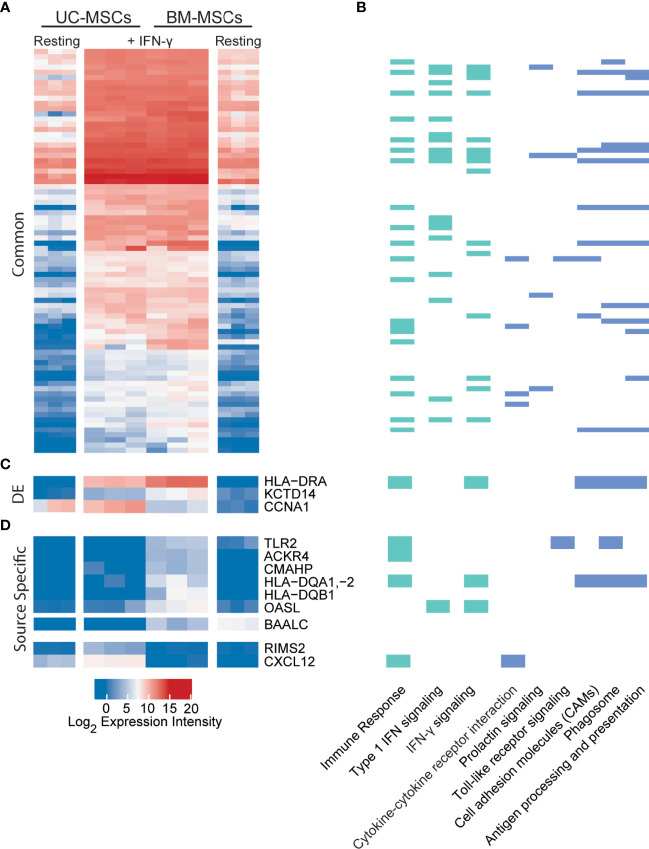
UC and BM-MSCs exhibit robust and highly similar transcriptome responses to to IFN-γ. **(A)** Seventy-eight DE genes were significantly upregulated in both MSC types by IFN-γ activation. **(B)** IFN-γ activation results in functional enrichment of immune and IFN signaling GO processes (green), among others (see complete list in **Table S4**), and 6 KEGG pathways (blue). **(C)** Three genes were upregulated by both UC and BM-MSCs but to significantly different levels. **(D)** Nine genes were expressed in a source-specific manner. 6 were upregulated specifically in BM-MSCs, while 2 were solely increased in UC-MSCs. One gene, *BAALC*, was downregulated in BM-MSCs but remained undetected in UC-MSCs. BM, bone marrow; DE, differentially expressed; IFN, interferon; MSC, mesenchymal stromal cell; UC, umbilical cord.

Dynamic transcriptome changes in UC and BM-MSCs following IFN-γ exposure also resulted in a striking convergence of transcriptome profiles (**Figure 4A**), leaving only 3 (*BAALC, CCNA1* and *CXCL12*) of the 174 DE genes between unstimulated MSC types significantly different (**Table 1**). Many responsive genes contribute to enriched KEGG pathways for antigen processing and presentation (15 genes) and cell adhesion molecules (10 genes), among others (**Figure 4B**). The top 5 enriched GO terms for this group of genes were the type I IFN signalling pathway (18 genes), IFN-γ-mediated signaling pathway (16 genes), immune response (21 genes), defense response to virus (14 genes) and antigen processing and presentation (9 genes) (**Figure 4B** and **Table S4**). In both MSC types, IFN-γ significantly induced *IDO1*, genes encoding MHC-II chain paralogues (*HLA-DRA*, *-DRB1/4/5*, *-DMA, -DMB*) and MHC-associated *CIITA*, as well as increased expression of *HLA-B/C/E/F* (MHC-I) and *BTN3A1* (**Table S5**). Notably, TNF-α and IL-1β did not have this effect. The only significantly downregulated transcript common to both stimulated MSC types was *GLS*, which encodes glutaminase. (**Table S5**).

Nine source-specific DE genes emerged as a consequence of IFN-γ polarization (**Table 1** and **Figures 4C, D**). Expression of *HLA-DRA* and *KCTD14* and *OASL* increased more substantially in BM-MSCs compared to UC-MSCs (**Figures 4C, D**), while *TLR2*, *ACKR4*, *CMAHP*, *HLA-DQA1, -2* and *HLA-DQB1* were specifically induced in BM-MSCs (**Figure 4D**). Stimulated UC-MSCs produced significantly higher levels of *RIMS2 and CXCL12*, which were undetected in both resting and activated BM-MSCs (**Figure 4D**). Overall, a small panel of DE genes predominantly corresponding to immunogenic cell surface proteins emerged as a BM-MSC signature, while UC-MSC uniquely express *CXCL12* and *RIMS2*.

### Confirmation of Array Data by RT-qPCR

Transcript levels for select genes of interest were validated using RT-qPCR (**Supplementary Figures 1A, B**). Consistent with the array data, *IDO1* was detected in only one resting UC-MSC population and was upregulated in all IFN-γ activated MSCs. Specific *HLA-DRA* induction by IFN-γ was also verified by RT-qPCR; *HLA-DRA* was also confirmed at higher levels in IFN-γ activated BM-MSCs. RT-qPCR also verified the source-specific regulation of *CXCL12* revealed by microarray; *CXCL12* transcript was more abundant in unstimulated UC-MSCs, upregulated in IFN-γ polarized UC-MSCs, and downregulated in TNF-α or IL-1β polarized UC-MSCs. *PTGS2* also proved to be specifically expressed by unstimulated UC-MSCs, and ubiquitously upregulated in response to all 3 cytokines although IL-1β elicited the strongest response. *TNFAIP6* (TSG-6), which is important for MSC immunosuppressive activity [65,66], was found to be modestly increased in response to IL-1β and IFN-γ and substantially upregulated in TNF-α activated BM-MSCs by microarray and RT-qPCR.

### Unstimulated and Activated UC and BM-MSCs Exhibit Unique Chemokine and Cytokine Secretion Profiles

The secreted protein profiles of cytokine-activated UC and BM-MSCs was next queried using a panel of 30 human cytokines and growth factors. The UC-MSCs proliferated faster than the BM-MSCs during the 24-hour activation period (**Supplementary Figure 2A**), so analyte concentration was normalized to the number of cells quantified at sample collection. The licensing cytokines were not detected in unconditioned media controls after the 24-hour incubation period (**Supplementary Figure 2B**). Experiments were performed in a protein-free media formulation to minimize confounding effects from background levels or signalling interactions caused by growth factors present in expansion media.

**Figure 5:**
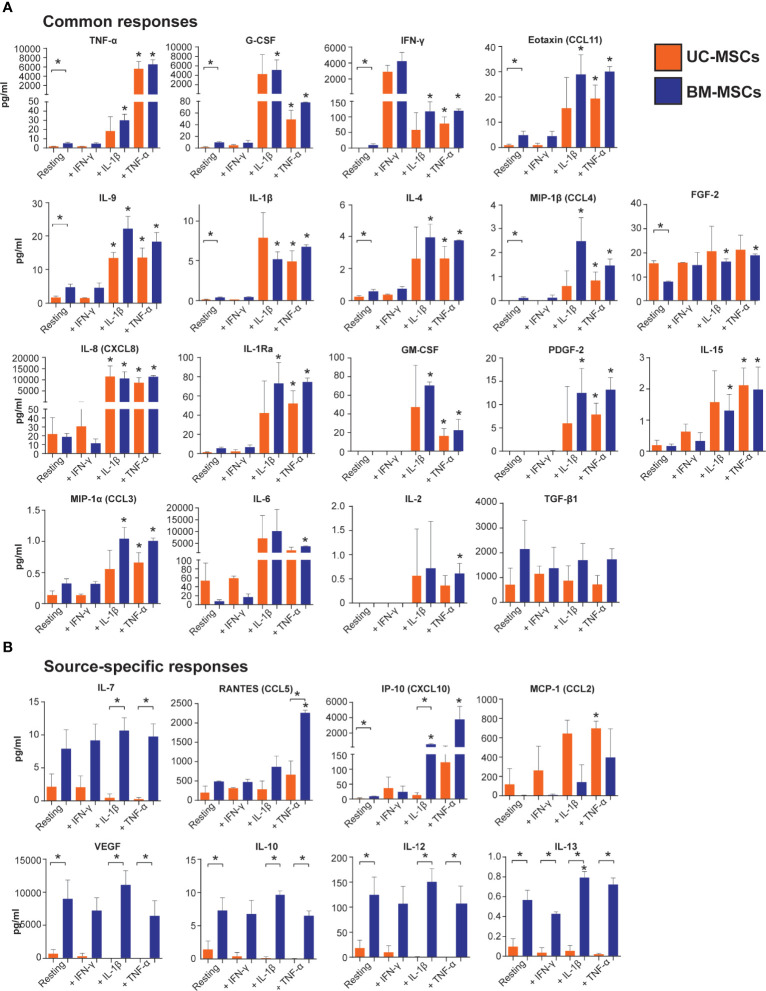
Output of inflammatory mediators by licensed UC and BM-MSCs has some source-specific differences. **(A)** Eighteen proteins exhibited similar expression patterns in unstimulated and licensed MSCs. **(B)** Nine proteins displayed source-specific expression patterns. Seven proteins were specifically produced at significantly higher concentrations by licensed BM-MSCs, while MCP-1 was uniquely upregulated by activated UC-MSCs. Protein concentration was normalized to per million cells at harvest after 24-hour activation. Bracketed asterisks denote significantly different means between MSC types, and unbracketed asterisks denote significantly different means between activated and resting MSCs of the same type, as determined by FDR-adjusted p<0.05. BM, bone marrow; MSC, mesenchymal stromal cell; UC, umbilical cord.

Unstimulated UC and BM-MSCs secreted substantial levels of TGF-β1 and RANTES and did not produce GM-CSF, IL-2, or PDGF-2 until stimulated (**Figure 5**). TGF-β2 and IL-17 were similarly expressed by resting and stimulated UC and BM-MSCs, although substantial donor variability for these analytes confounded statistical analysis (**Supplementary Figure 2C**). TGF- β3 and IL-5 were never detected (not shown).

Thirteen cytokines were more abundantly secreted (p<0.05) by unstimulated BM-MSCs (**Table S6** and **Figure 5**), including VEGF, IL-12, G-CSF and IP-10. Nine other cytokines, IL-10, IL-13, TNF-α, IFN- γ, eotaxin, IL-9, IL-1β, IL-4, and MIP-1β, were secreted at low concentrations (<10 pg/ml/million cells) by BM-MSCs, but ranged from undetectable to <2 pg/ml/million cells in CM from UC-MSCs (**Table S6** and **Figure 5**). Unstimulated UC-MSCs secreted more FGF-2 (1.9-fold higher, p<0.05) than BM-MSCs, and more IL-6, IL-8 and MCP-1 albeit at non-significant levels (p>0.05) due to donor variability (**Figure 5** and **Table S6**).

TNF-α elicited a significant secretory response from both UC and BM-MSCs. Production of 17 soluble proteins increased from both MSC types. Final concentrations of TNF-α, G-CSF, IFN-γ, eotaxin, IL-9, IL-1β, IL-4, MIP-1β and FGF-2 were similar between UC and BM-MSCs (**Figure 5** and **Table S7**). By contrast, TNF-α activated BM-MSCs secreted significantly higher amounts of IL-7 and RANTES, which UC-MSCs significantly reduced IL-7 synthesis (**Table 1** and **Figure 5**). TNF-α did not modulate IL-10, IL-12, IL-13 or VEGF output from either cell type; these remained more highly secreted by BM-MSCs (**Figure 5**, **Table 1** and **Table S7**).

IL-1β stimulated production of analytes similar to TNF-α (**Figure 5**). However, we documented substantial donor variability in protein-level responses to IL-1β which was not evident on the gene level or in response to TNF-α or IFN-γ. Interestingly, UC-MSCs reduced IL-7 and IP-10 output in response to IL-1β, while BM-MSCs substantially increased secretion of these proteins (**Figure 5** and **Table S7**).

IFN-γ had little detectable effect on the selected panel of pro-inflammatory cytokines (**Figure 5**). Both UC and BM-MSCs substantially increased IFN-γ secretion via positive feedback (**Supplementary Figure 2B** and **Figure 5**). IP-10 also increased >2-fold in both IFN-γ activated MSC types but did not meet statistical significance criteria (**Figure 5** and **Table S7**). IL-13 was found to be significantly different between IFN-γ activated MSC types, but all measured CM concentrations were <1pg/ml (**Figure 5**).

### Gene and Protein Expression Profiles Are Not Consistent

We next assessed the correlation between gene expression and protein output. Of the assayed proteins, concomitant abundance of transcripts after licensing were evident for IL-6, IL-8, VEGF, FGF-basic, MCP-1 and TGF- β1 (**Supplementary Figure 3**). Others, like RANTES, were only correlative in a subset of activating conditions. This data shows that only a subset of gene and protein biomarkers of MSC activation can be used interchangeably.

## Discussion

Tissue and donor-influenced variability between MSC populations has been well established (9–11, 14–19). However, phenotypic and functional assessments have predominantly been performed on unstimulated cells (12, 69–72). Once administered to a patient, MSCs respond to the wounded or diseased environment and become polarized, adopting an activated phenotype. Van Megen et al. reported that the surface profiles of BM-MSCs still meet MSC minimal criteria after activation by IFN-γ (73). We also found that activated phenotype of MSCs did not influence MSC surface marker profiles (data not shown). We postulated that evaluation of polarized MSC populations after cytokine activation might provide important insights into their functional efficacy and inform development of cellular therapies and quality control assays across donors and tissue of origin.

Our data show remarkable synchronization of UC and BM-MSC transcriptomes upon activation with each of the selected cytokines, although responses to each cytokine were different. Multiple trajectories of change in gene expression markedly resolved the heterogeneity between unstimulated cell populations. These transcriptome shifts suggest that polarized MSCs may share many fundamental mechanisms of action. Consistent with this notion, Szabó et al. reported that IFN-γ and TNF-α pre-conditioning synchronized murine MSCs and attenuated donor-imprinted functional heterogeneity (73). Pre-activation of MSCs has been shown to improve therapeutic potency and reproducibility (5, 27, 74–81), to restore functionality of senescent cells (82), and to support context-dependent recovery of functional cells from cryopreservation (83, 84). The reported variable outcomes in manufacturing and therapeutic success between MSC sources (8, 69, 70, 72) may therefore be the consequence of a small cohort of functionally relevant factors differentially expressed after licensing or *in vivo* deployment, rather than the non-specific heterogeneity found in unstimulated cells. Conversely, controlled stimulation with selected cytokines or other defined stressors could be leveraged to strategically manipulate specific therapeutic properties of MSCs.

TNF-α stimulation revealed a cohort of genes involved in viral immunity and the Type 1 IFN signalling pathway that were specifically upregulated in activated BM-MSCs, although they had been similarly expressed in resting UC and BM-MSCs. In addition, *bone marrow stromal antigen 2* (*BST2;* tetherin), *matrix metalloprotease 3* (*MMP3)* and *signal transducer and activator of transcription 4* (*STAT4)* were DE by BM-MSCs after TNF-α and IL-1β activation, suggesting that they may be functional mediators important to BM-specific mechanisms of action. By contrast, *G-protein coupled receptor family C group 5 member B* (*GPRC5B)* and *mitogen-activated protein kinase kinase kinase 8* (*MAP3K8)* were reproducibly DE by UC-MSCs in response to TNF-α and IL-1β. Intriguingly, *regulating synaptic membrane exocytosis protein 2* (*RIMS2*) was only induced in UC-MSCs following IFN-γ exposure, while several human leukocyte antigen (HLA) receptors, involved in antigen presentation, and *toll like receptor 2* (*TLR2*), which recognizes pathogenic peptides, were specifically upregulated in BM-MSCs.

*CXCL12* emerged as a dynamic signature gene unique to UC-MSCs. *CXCL12* was expressed by unstimulated UC-MSCs, but not BM-MSCs. Upon activation by TNF-α and IL-1β, expression intensity of *CXCL12* decreased, most substantially in response to IL-1β, and remained off in BM-MSCs. IFN-γ stimulated significant upregulation of *CXCL12* from UC-MSCs, and a mild upregulation from BM-MSCs, but only to within the detectable range. *CXCL12* encodes Stromal Derived Factor (SDF-1), a chemotactic molecule for lymphocytes and MSCs. We have documented that UC-MSCs increase SDF-1 production in response to full-thickness burns (Braid et al, 2022, in prep), supporting the notion that *CXCL12* is an important factor relevant to therapeutic efficacy and potentially UC-MSC-specific treatment outcomes.

The data also provide evidence that MSCs from different sources may achieve similar functional outcomes by different mechanisms. MCP-1, VEGF, and IL-6 are important for MSC-mediated angiogenesis (85), in addition to other functions. In this study, unstimulated BM-MSCs produced significantly higher amounts of VEGF, while resting UC-MSCs produced higher amounts of MCP-1 and IL-6. Thus, CM isolated from either unstimulated MSC type possesses pro-angiogenic factors of different identity. Following TNF-α or IL-1β activation, however, BM-MSCs and UC-MSCs secreted comparable levels of IL-6, while UC-MSCs produced more MCP-1 than BM-MSCs which exhibited donor-influenced increases in MCP-1 output. By contrast, VEGF production by either MSC type was largely unaffected by any priming condition. The functional efficacy of the activated cells and resulting CM produced in this study are being evaluated to better understand the functional relationship between the changes documented here.

The current standard for establishing immune-modulatory potency of MSCs is the T-cell suppression assay, in which MSCs are co-cultured with lymphocytes and reduced T-cell proliferation correlates to MSC potency. Chinnadurai et al. identified a panel of genes upregulated by both IFN-γ activated MSCs and MSCs exhibiting immune-suppressive activity in T-cell suppression assays (86). Chinnadurai’s study suggests that a simplified assay for MSC immunosuppressive potency or activation status is possible, since IFN-γ activation can be used as a surrogate model for the more complex and costly co-culture assay (86). Here, IFN-γ evoked highly similar and convergent transcriptome responses from UC and BM-MSCs, supporting IFN-γ activation as a surrogate assay applicable to multiple MSC types. The lack of secretory response to IFN-γ found here is likely a consequence of analyte selection, since many of the hallmark IFN-γ responsive proteins were not represented on the panel.

IFN-γ-mediated activation has also been proposed as a “universal” surrogate to assess general MSC potency (1). However, our study revealed minimal overlap in secreted markers between the TNF-α, IL-1β and IFN-γ activation states. We also noted that 1 population each of UC-MSCs and BM-MSCs were significantly more responsive to IL-1β than the other populations tested. Interestingly, these 2 populations did not exhibit such sensitivity to either TNF-α or IFN-γ. Redondo-Castro et al. reported that IL-1β stimulated higher levels of IL-6 and G-CSF from BM-MSCs than TNF-α or IFN-γ (87). We documented the same pattern here, for both UC and BM-MSCs. In Redondo-Castro’s study, CM from IL-1β-activated BM-MSCs reduced synthesis of pro-inflammatory cytokines secreted by inflamed microglial cells via G-CSF, while CM from TNF-α or IFN-γ-licensed BM-MSCs did not (87). These findings suggest that MSC responses to IFN-γ alone may not adequately predict responsiveness to other activation pathways or mechanisms of action, and support development of potency assays based on the MSCs’ proposed utility (8).

Differences in the fundamental properties of UC and BM-MSCs make certain comparisons challenging. For example, the doubling time of UC-MSCs is shorter than BM-MSCs (19, 71, 88). Although cells were seeded at the same density in our experiments, significantly more UC-MSCs were harvested 36 hours later, at the time of collection. Raw data from the CM analysis showed that analyte concentrations in the UC-MSC CM were substantially higher than in CM from BM-MSCs. However, once the results were normalized to the number of cells at the time of collection, resting and activated BM-MSCs emerged as more substantial producers of many of the soluble mediators analyzed. A recent study showed that UC-MSCs are more responsive than BM-MSCs to TNF-α stimulation (89). In that study, UC-MSCs rapidly produced TSG-6, and then TSG-6 expression tapered off over a 24-hour period. Conversely, secretion of TSG-6 from BM-MSCs took longer to initiate and peaked at 24 hours. Thus, it is difficult to extrapolate the functional relevance of absolute analyte values documented in this study which may vary depending on the sampling time point. However, clear trends emerged from the data. VEGF, TGF-β1, IL-7, IL-10, IL-12 and IL-13 were consistently secreted at higher protein to cell ratios by BM-MSCs, independent of priming condition. Moreover, secretion of these factors was mainly unaffected by any of the priming conditions for both MSC types. With the exceptions of IP-10, RANTES and MCP-1, the soluble responses of UC and BM-MSCs were similar in both identity and amplitude for the remaining analytes.

Our results suggest that assessments of variability between unstimulated MSCs or resting and polarized counterparts may not be fully predictive of tissue or donor-imprinted differences in therapeutic efficacy or mechanisms of action. *IDO1* is an accepted surrogate marker of immunosuppressive activity for MSCs. IFN-γ stimulates *IDO1* expression, and the degree of responsiveness to IFN-γ purportedly predicts MSC immune-modulatory potency (90). Using this paradigm, however, one potent UC-MSC population would have been discarded as “unresponsive”. Unlike the other MSC populations used in this study, unstimulated cells from this UC-MSC donor robustly expressed *IDO1*. In response to IFN-γ, the other MSC populations increased *IDO1* to levels that matched the pre-stimulation *IDO1* levels from this donor population. Evaluation of the pre-activation or resting state would have predicted this donor population to have superior immunosuppressive function, when in fact it generated comparable *IDO1* levels in response to all the IFN-γ activated MSC. Conversely, fold-change metrics would have marked this population as an unsuitable non-responder.

Development of reliable assays to qualify MSCs based on identity and functional utility have been hindered by the overwhelming heterogeneity of MSC populations derived from different donors and tissues. The remarkable transcriptome convergence documented in this study implies that a substantial proportion of the heterogeneity in unstimulated cells may simply be noise (70). Once activated, this background is resolved and the meaningful differences between MSC populations emerge. We propose that focused assessment of activated MSC phenotypes can refine and expedite the development of robust surrogate assays and release criteria that clearly distinguish MSC populations with different functional properties.

## Data Availability Statement

The datasets presented in this study can be found in online repositories. The names of the repository/repositories and accession number(s) can be found in: NCBI’s Gene Expression Omnibus (GEO), accession number GSE129165.

## Author Contributions

DW: Collection and/or assembly of data, data analysis and interpretation, manuscript writing, final approval of the manuscript. CW: Collection and/or assembly of data, final approval of the manuscript. BF: Financial support, final approval of the manuscript. LB: Conception and design, collection and/or assembly of data, data analysis and interpretation, manuscript writing, final approval of the manuscript.

## Funding

This work was funded by Public Service and Procurement Canada contracts W7702 175853 and W7714 196914 to Aurora BioSolutions Inc.

## Conflict of Interest

LB is an officer and a shareholder of Aurora BioSolutions Inc. DW and CW are employees of Aurora BioSolutions Inc.

The remaining authors declare that the research was conducted in the absence of any commercial or financial relationships that could be construed as a potential conflict of interest.

## Publisher’s Note

All claims expressed in this article are solely those of the authors and do not necessarily represent those of their affiliated organizations, or those of the publisher, the editors and the reviewers. Any product that may be evaluated in this article, or claim that may be made by its manufacturer, is not guaranteed or endorsed by the publisher.

## Supplementary Material

Supplementary Figure 1
**(A)** Primer sequences used in RT-qPCR for 3 reference genes and 5 genes of interest. **(B)** Relative expression of genes of interest found by RT-qPCR, displayed as mean ± SEM of 3 donors, confirms their microarray expression. BM, bone marrow; F, forward; MSCs, mesenchymal stromal cells; R, reverse; ref, reference gene; UC, umbilical cord.

Supplementary Figure 2
**(A)** UC and BM-MSC donor populations exhibit different doubling kinetics during the activation and CM collection period. Secreted analyte concentration was normalized to cell number at harvest and analyzed using units of pg/ml/million cells. **(B)** Dosed cytokines were not detected in UM after the 24-hour activation period. **(C)** Substantial MSC donor variability in secretion of TGF-β2 and IL-17 precluded statistical analysis. BM, bone marrow; CM, conditioned media; MSC, mesenchymal stromal cell; UC, umbilical cord; UM, unconditioned media.

Supplementary Figure 3 Correlation between transcriptome and secreted responses of resting and activated UC (orange) and BM-MSCs (blue). **(A)** A modest relationship exists between transcriptome expression and secreted protein concentration in CM from resting MSCs. When MSCs are primed with **(B)** TNF-α, **(C)** IL1-β or **(D)** IFN-γ, substantial changes in protein expression are often not coupled to a measured increase in corresponding transcript abundance and vice versa. BM, bone marrow; CM, conditioned media; MSC, mesenchymal stromal cell; UC, umbilical cord.
