# Supplementary figures and images for: Cytokine Activation Reveals Tissue-Imprinted Gene Profiles of Mesenchymal Stromal Cells
Source: Front Immunol. 2022 Jul 18;13:917790. doi: 10.3389/fimmu.2022.917790 (PMC9341285; doi:10.3389/fimmu.2022.917790)

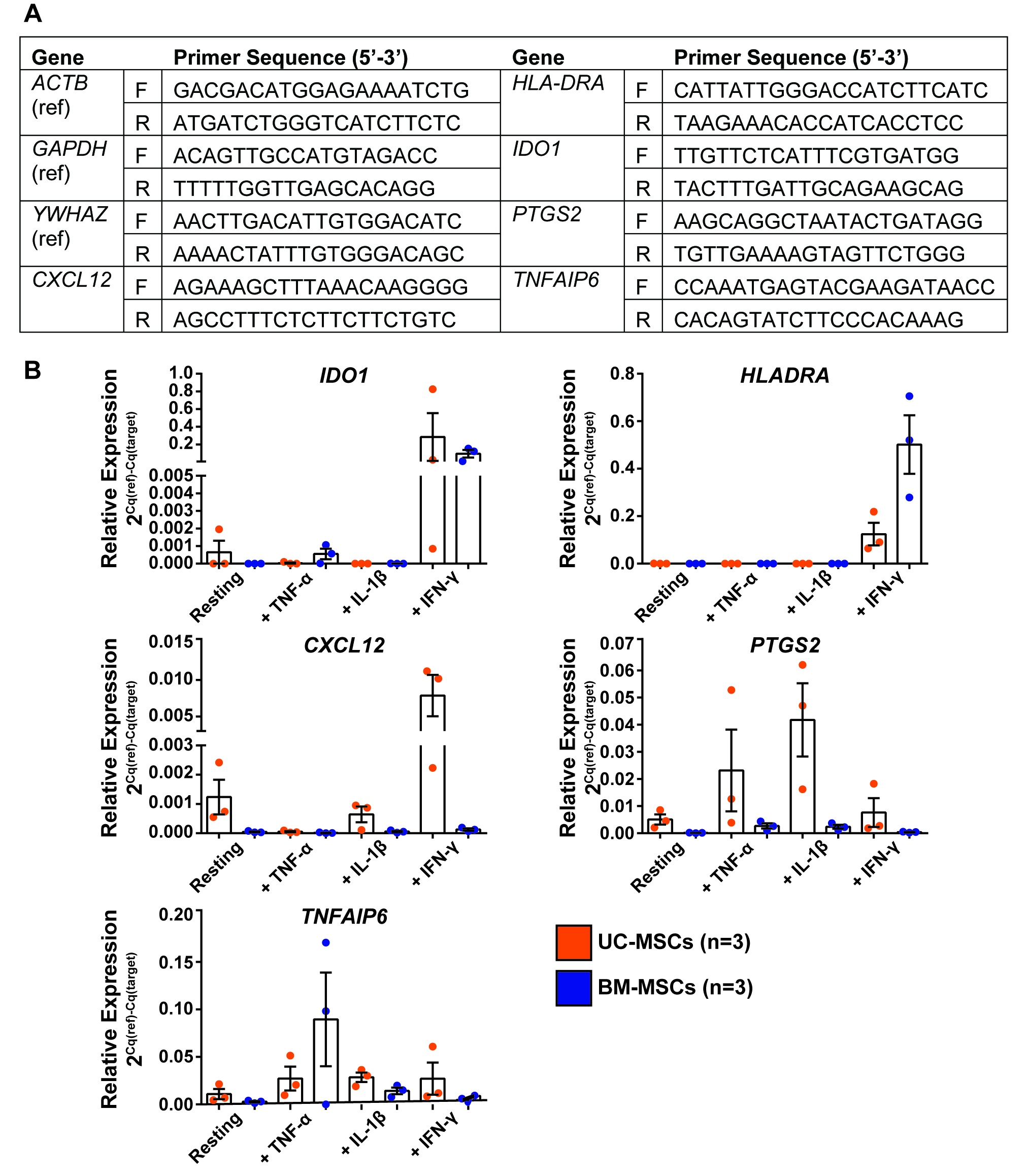

Supplement: Supplementary Figure 1 — (A) Primer sequences used in RT-qPCR for 3 reference genes and 5 genes of interest. (B) Relative expression of genes of interest found by RT-qPCR, displayed as mean ± SEM of 3 donors, confirms their microarray expression. BM, bone marrow; F, forward; MSCs, mesenchymal stromal cells; R, reverse; ref, reference gene; UC, umbilical cord. [file Image_1.tif]

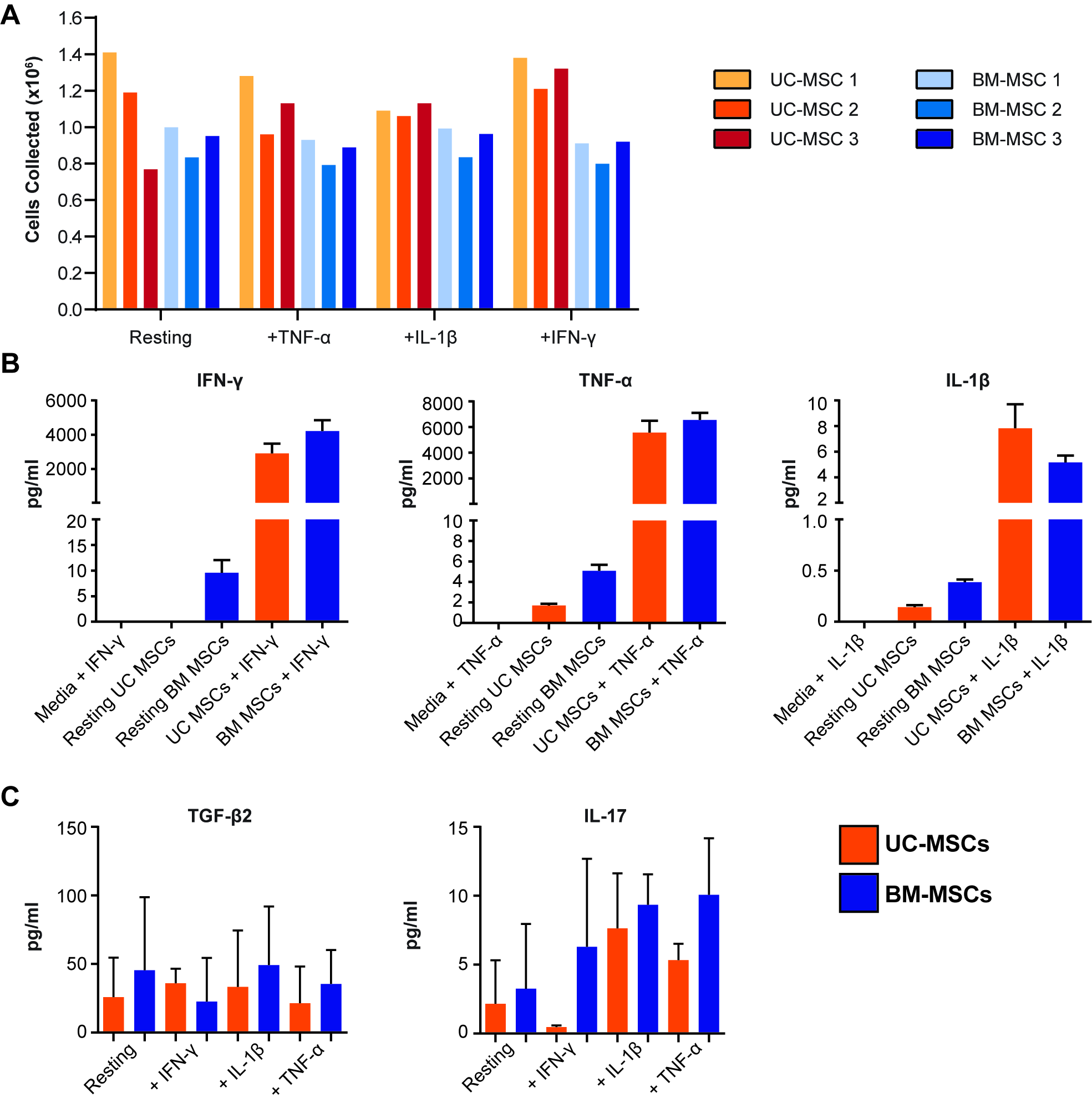

Supplement: Supplementary Figure 2 — (A) UC and BM-MSC donor populations exhibit different doubling kinetics during the activation and CM collection period. Secreted analyte concentration was normalized to cell number at harvest and analyzed using units of pg/ml/million cells. (B) Dosed cytokines were not detected in UM after the 24-hour activation period. (C) Substantial MSC donor variability in secretion of TGF-β2 and IL-17 precluded statistical analysis. BM, bone marrow; CM, conditioned media; MSC, mesenchymal stromal cell; UC, umbilical cord; UM, unconditioned media. [file Image_2.tif]

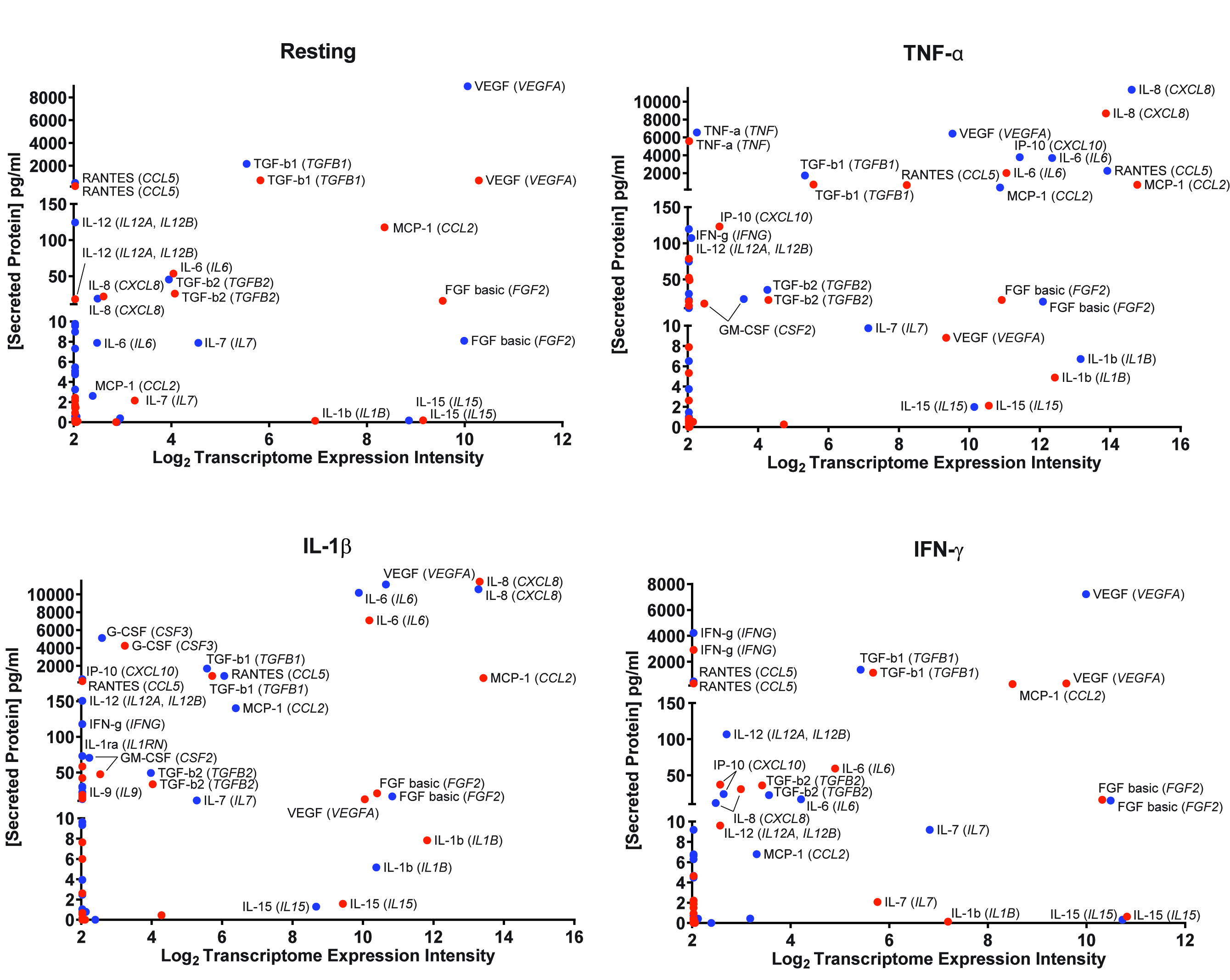

Supplement: Supplementary Figure 3 — Correlation between transcriptome and secreted responses of resting and activated UC (orange) and BM-MSCs (blue). (A) A modest relationship exists between transcriptome expression and secreted protein concentration in CM from resting MSCs. When MSCs are primed with (B) TNF-α, (C) IL1-β or (D) IFN-γ, substantial changes in protein expression are often not coupled to a measured increase in corresponding transcript abundance and vice versa. BM, bone marrow; CM, conditioned media; MSC, mesenchymal stromal cell; UC, umbilical cord. [file Image_3.tif]
